# Biodiversity of Environmental *Leptospira*: Improving Identification and Revisiting the Diagnosis

**DOI:** 10.3389/fmicb.2018.00816

**Published:** 2018-05-01

**Authors:** Roman Thibeaux, Dominique Girault, Emilie Bierque, Marie-Estelle Soupé-Gilbert, Anna Rettinger, Anthony Douyère, Michael Meyer, Gregorio Iraola, Mathieu Picardeau, Cyrille Goarant

**Affiliations:** ^1^Leptospirosis Research and Expertise Unit, Institut Pasteur in New Caledonia, Institut Pasteur International Network, Noumea, New Caledonia; ^2^Bacteriology and Mycology, Institute for Infectious Diseases and Zoonoses, Department of Veterinary Sciences, Faculty of Veterinary Medicine, Ludwig Maximilian University of Munich, Munich, Germany; ^3^Institut des Sciences Exactes et Appliquées, Plateau MET/MEB, Université de la Nouvelle-Calédonie, Noumea, New Caledonia; ^4^Bioinformatics Unit, Institut Pasteur Montevideo, Montevideo, Uruguay; ^5^Biology of Spirochetes Unit, Institut Pasteur, National Reference Centre and WHO Collaborating Center for Leptospirosis, Paris, France

**Keywords:** soil microbiology, MALDI-TOF MS, WGS comparisons, novel species, isolation

## Abstract

Leptospirosis is an important environmental disease and a major threat to human health causing at least 1 million clinical infections annually. There has recently been a growing interest in understanding the environmental lifestyle of *Leptospira*. However, *Leptospira* isolation from complex environmental samples is difficult and time-consuming and few tools are available to identify *Leptospira* isolates at the species level. Here, we propose a polyphasic isolation and identification scheme, which might prove useful to recover and identify environmental isolates and select those to be submitted to whole-genome sequencing. Using this approach, we recently described 12 novel *Leptospira* species for which we propose names. We also show that MALDI-ToF MS allows rapid and reliable identification and provide an extensive database of *Leptospira* MALDI-ToF mass spectra, which will be valuable to researchers in the leptospirosis community for species identification. Lastly, we also re-evaluate some of the current techniques for the molecular diagnosis of leptospirosis taking into account the extensive and recently revealed biodiversity of *Leptospira* in the environment. In conclusion, we describe our method for isolating *Leptospira* from the environment, confirm the usefulness of mass spectrometry for species identification and propose names for 12 novel species. This also offers the opportunity to refine current molecular diagnostic tools.

## Introduction

Leptospirosis is a bacterial disease that affects human and animals that are exposed to a contaminated environment; usually water or soils contaminated by the urine of reservoir animals. The global burden of leptospirosis is estimated to more than 1 million cases and 60,000 fatalities annually, mostly impacting rural or suburban impoverished populations in tropical and subtropical regions worldwide (Costa et al., 2015).

Leptospires are thin helical-shaped bacteria from the phylum of *Spirochaetes*. One century ago, freshwater saprophytic leptospires and pathogenic leptospires, the etiological agents of leptospirosis, had been first described and rats had been identified as the main animal reservoirs of virulent strains (Ido et al., 1917; Noguchi, 1917). Prophetically, in the same issue of The Journal of Experimental Medicine, Noguchi questioned the survival of leptospires in nature, including in water and soils, and studied the interactions of *Leptospira* with other bacteria (Noguchi, 1918a). The foundations of the epidemiology and taxonomy of *Leptospira* were therefore clearly established in a very short period of time one century ago (Noguchi, 1918b).

Pathogenic *Leptospira* have then been classified as *L. interrogans sensu lato* while saprophytes were named *L. biflexa sensu lato* for decades (both denominations being now inappropriate in modern taxonomy), until the 1980's and the description of *Leptospira inadai* (Schmid et al., 1986). This species later proved to be the first representative of the so-called “intermediate” cluster (Hookey et al., 1993). At the end of the twentieth century, there were 16 validly described *Leptospira* species, with the description of a second intermediate species *Leptospira fainei* (Perolat et al., 1998) and further molecular reclassification of collection strains (Brenner et al., 1999).

In the recent years, a pan-*Leptospira* genome project supported by the National Institute of Allergy and Infectious Disease (NIAID) Genome Sequencing Center has provided the scientific community with hundreds of whole-genome sequences allowing large comparative genomic analyses (Lehmann et al., 2013, 2014; Fouts et al., 2016). This has offered new insights into the phylogeny of the genus *Leptospira*, notably confirming the discrimination of the three major clades (“pathogens,” “intermediates,” and “saprophytes”) by whole-genome analysis. Within the pathogens, this has also allowed to identify sub-clusters of varying pathogenicity (Xu et al., 2016). Our recent genomic study of environmental isolates from New Caledonia, a tropical island where leptospirosis is endemic, suggested that more heterogeneity in virulence exists in the cluster named “pathogens” and that virulence was acquired independently in “pathogens” and “intermediates” (Thibeaux et al., 2018). However, major questions are still open about the comparative virulence (Picardeau, 2017) as some species from the cluster “intermediates” are increasingly recognized in human disease (Tsuboi et al., 2017; Puche et al., 2018).

In this study, we isolated *Leptospira* from soils suspected to be contamination sources in New Caledonia. Isolates were submitted to a polyphasic approach for identification, with three sequential steps. First, a melting curve analysis of the 16S ribosomal RNA gene achieved a fast presumptive identification of the cluster (i.e., pathogens, intermediates, or saprophytes). A proteomic analysis based on matrix-assisted laser desorption ionization time-of-flight (MALDI-ToF) mass spectrometry using the MALDI Biotyper Systems allowed species identification through pairwise comparison with reference spectra of the 23 validly described *Leptospira* species. Whole-genome sequencing (WGS) for formal identification of all 26 isolates provided unambiguous identification of clusters and species, thus allowing to evaluate the relevance of the identification scheme. In total, whole-genome sequencing confirmed the relevance of this approach and showed that MALDI-ToF spectra-based identification is a powerful tool to identify *Leptospira* at the species level. In parallel, the biodiversity revealed from these environmental isolates also questions molecular diagnosis. Therefore, we evaluated two real time PCR assays for leptospirosis diagnosis (Merien et al., 2005; Stoddard et al., 2009) and discuss possible improvement as well as their applicability in different contexts.

## Materials and methods

### *Leptospira* reference strains and environmental isolates

*Leptospira* strains used in this study (Supplementary Table 1) were from the bacterial collections of the Institut Pasteur in New Caledonia and in Paris.

**Figure 1:**
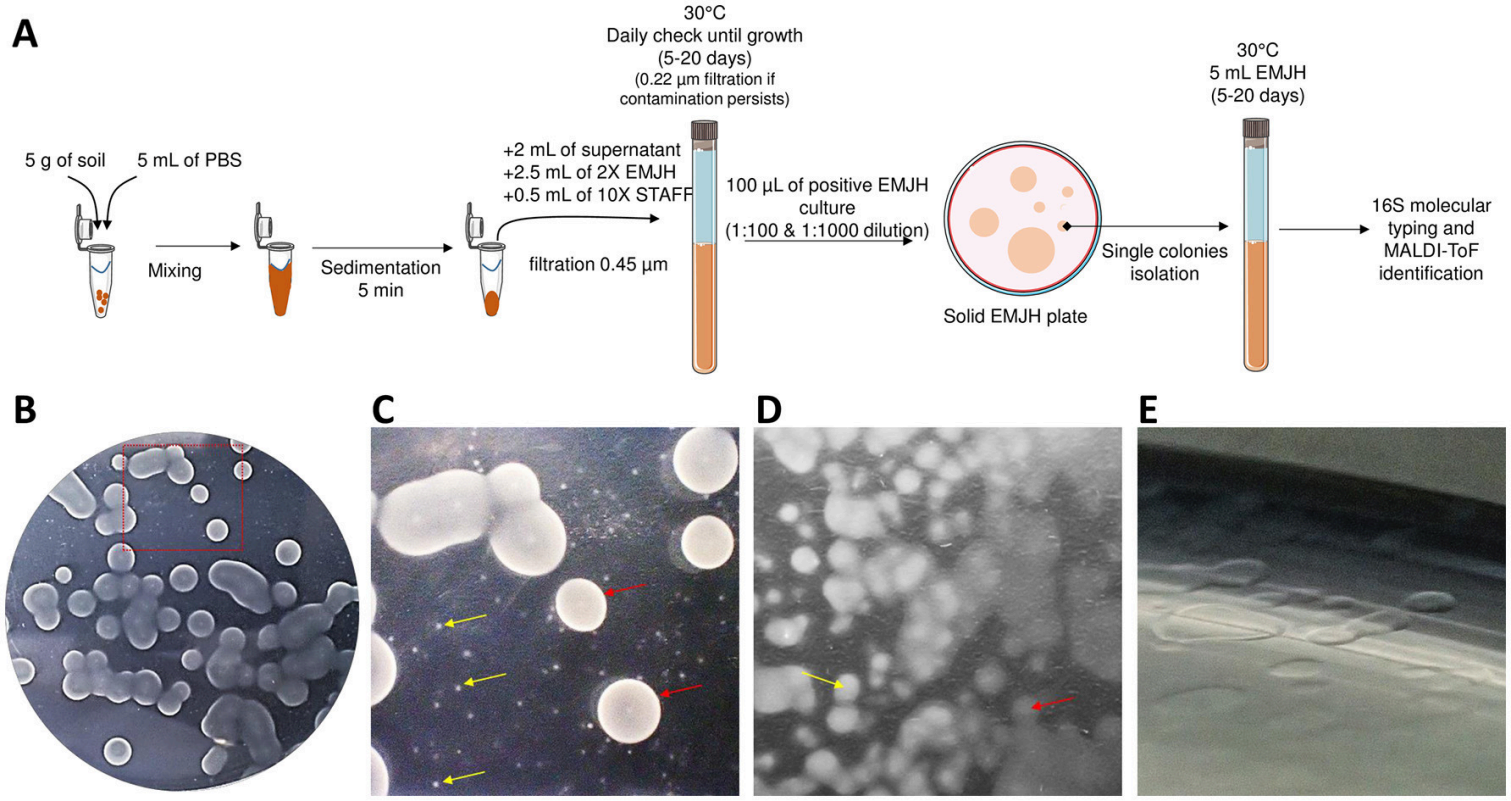
*Leptospira* isolation on EMJH agar plates. **(A)** Global isolation strategy. **(B)** Macroscopic aspect of colonies. **(C)** Closer view from **(A)** showing colonies of different sizes. **(D)** Opaque (yellow arrow) and transparent (red arrow) colonies. **(E)** Translucent colonies.

Environmental isolates as well as isolation procedure have been described previously (Thibeaux et al., 2018). Briefly, 5 g of topsoil were mixed with 5 mL of PBS (phosphate buffered saline) and 2 mL of supernatant were filtered through a sterile 0.45 μm filter into a tube filled with 2.5 mL of Ellinghausen-McCullough-Johnson-Harris (EMJH) medium 2x and 0.5 mL of 10X concentrated STAFF (sulfamethoxazole, trimethoprim, amphotericin B, fosfomycin, and 5-fluorouracil) (Chakraborty et al., 2011). Cultures were incubated at 30°C and when spirochetes were observed, 100-μL of the diluted culture (1:100 and 1:1,000) was plated onto solid EMJH agar (12 g/L) using sterile glass beads and incubated at 30°C until individual subsurface colonies were visible. One to five characteristic subsurface individual colonies from each plate were collected with a sterile tip, for confirmation of a *Leptospira* morphology and motility by dark field microscopy before clonal subculture in liquid EMJH. The isolation procedure is summarized in Figure 1A.

### DNA extraction and 16S RNA molecular typing

Genomic DNA was extracted from 200 μL of EMJH liquid clonal cultures. Extraction was performed using a commercial kit (QIAamp® DNA Mini Kit, Qiagen, Australia) according to the manufacturer's instructions. DNA elution was performed with 50 μL of buffer AE. The quantity of DNA was measured with NanoDrop™ (Thermo Fisher Scientific, Waltham, MA USA) and adjusted to 0.1 ng/μL.

Amplification of a fragment of the 16S rRNA gene was performed in a total volume of 10 μL on a LightCycler 480 II. The reaction mixture consisted in 2 μL PCR grade water, 5 μL of 2X reaction SYBR Green Mix (LC480 SYBR Green I Master kit, Roche, New Zealand), 0.5 μL of forward primer (5′-GGCGGCGCGTCTTAAACATG-3′), 0.5 μL of reverse primer (5′-CTTAACTGCTGCCTCCCGTA-3′), and 2 μL (0.2 ng) of DNA (Mérien et al., 1992). After amplification, the melting temperature of the amplified product was analyzed using LightCycler 480 Software.

### MALDI-ToF mass spectrum analysis: sample preparation and construction of a database

Samples were prepared as described in an earlier study (Rettinger et al., 2012) with minor changes. Briefly, *Leptospira* cells from liquid EMJH culture were harvested by centrifugation (18,000 g for 10 min) of 1 mL of an exponential 8-day old culture in EMJH culture medium. The pellet was resuspended in 200 μL PBS to wash cells and remove the excess of protein coming from the EMJH culture medium. Bacterial cells were pelleted again by identical centrifugation (18,000 g for 10 min). The pellet was resuspended and homogenized in 300 μL sterile distilled water before addition of 900 μL absolute ethanol. At this step, the sample can be stored up to 6 months at −20°C. The cells were pelleted again (18,000 g for 10 min), the pellet was dried under a hood. Finally, the dry pellet was thoroughly homogenized in 30 μL 70% formic acid before addition of 30 μL pure acetonitrile.

The homogeneous mixture was centrifuged 2 min at 15,000 g and 1 μL of the supernatant was spotted on a MSP-96 polished steel target plate (Bruker Daltonics, Wissembourg, France) and allowed to dry at room temperature. The sample was overlaid with 1.2 μL matrix (10 mg α-cyano-4-hydroxy-cinnamic acid / mL in 50% acetonitrile/2.5% trifluoroacetic acid). Mass spectra (MS) were acquired using a Microflex mass spectrometer (Bruker Daltonics, Wissembourg, France) in the linear mode and a mass range of 2–20 kDa using the automated functionality of flexControl 3.0 software (Bruker Daltonics, Wissembourg, France). To create reference spectra, at least 20 raw spectra (8 spots read 3 times each) were used to generate a main spectrum. A database was constructed with the reference MS of 43 strains (30 pathogenic, 6 intermediates and 7 saprophytes, Supplementary Table 1) encompassing representative of all 23 validly described species.

To identify isolates, samples were prepared similarly. The MS database was loaded into MALDI Biotyper 3.0 software (Bruker Daltonics) and used to compare the MS of the isolate with the MS database. The software MALDI Biotyper calculates a similarity score considering the proportion of matching peaks between the analyzed MS and all MS in the database. The similarity score values range from 0 (no similarity) to 3 (absolute identity). An identification score ≥2.3 was considered valid for identification at the species level as recommended by the manufacturer and confirmed in a previous study (Rettinger et al., 2012). We first loaded reference MS created in another laboratory (Rettinger et al., 2012) to evaluate the inter-laboratory portability of reference MS. Using this imported database, we submitted our routine strains and isolates (bold lines in Supplementary Table 1) to identification. We considered that an identification score in the range 2.0–2.3 could reveal “possible” novel species whereas a score below 2.0 was regarded as a “probable” novel species (Table 1). The main spectra library (MSP) dendrogram was created by the standard MALDI Biotyper MSP creation method (Bruker Daltonics, Bremen, Germany), where distance values are relative and are always normalized to a maximum value of 1,000.

**Table 1:** Summary of the stepwise identification process using 26 environmental isolates.

	***STEP I:*** **Clade identification**		***STEP II:*** **Maldi ToF identification**		***STEP III:* Identification through WGS**
**Isolate**	**16S rRNA T_m_**	**Clades**	**Maldi-ToF score**	**Maldi-ToF identification**	**Formal identification**
ATI2-C-A1	85.11	Saprophyte	1.93	*probable novel saprophyte*	*L. macculloughii* sp. nov. (sp. nov sapro 4)
FH2-B-B2	85.17	Saprophyte	1.88	*probable novel saprophyte*	*L. harrisiae* sp. nov. (sp. nov sapro 1)
MCA2-B-A1	85.17	Saprophyte	1.93	*probable novel saprophyte*	*L. levettii* sp. nov. (sp. nov sapro 2)
JW2-C-A2	85.20	Saprophyte	1.86	*probable novel saprophyte*	*L. brenneri* sp. nov. (sp. nov sapro 3)
CN6-C-A1	85.24	Saprophyte	1.90	*probable novel saprophyte*	*L. levettii* sp. nov. (sp. nov sapro 2)
CN1-B-A1	85.31	Saprophyte	1.97	*probable novel saprophyte*	*L. levettii* sp. nov. (sp. nov sapro 2)
ATI2-C-A2	85.31	Saprophyte	2.42	*L. meyeri*	*L. meyeri*
FH2-B-A1	85.33	Saprophyte	2.04	*possible novel saprophyte*	*L. harrisiae* sp. nov. (sp. nov sapro 1)
ES1-C-A1	85.37	Saprophyte	2.31	*L. meyeri*	*L. meyeri*
ES1-C-A2	85.40	Saprophyte	2.31	*L. meyeri*	*L. meyeri*
FH4-C-A1	85.81	Pathogenic	1.87	*probable novel pathogen*	*L. barantonii* sp. nov. (sp. nov patho 2)
ATI7-C-A5	85.84	Pathogenic	1.50	*probable novel pathogen*	*L. ellisii* sp. nov. (sp. nov. patho 1)
FH2-B-D1	85.92	Pathogenic	1.49	*probable novel pathogen*	*L. adleri* sp. nov. (sp. nov. patho 3)
FH2-B-C1	85.95	Pathogenic	1.33	*probable novel pathogen*	*L. adleri* sp. nov. (sp. nov. patho 3)
ATI7-C-A3	85.95	Pathogenic	1.41	*probable novel pathogen*	*L. ellisii* sp. nov. (sp. nov. patho 1)
JW2-C-B1	86.15	Pathogenic	2.41	*L. kmetyi*	*L. kmetyi*
JW3-C-A1	86.18	Pathogenic	2.42	*L. kmetyi*	*L. kmetyi*
MCA2-B-A3	86.52	Intermediate	1.82	*probable novel intermediate*	*L. hartskeerlii* sp. nov. (sp. nov inter 5)
FH4-C-A2	86.52	Intermediate	1.45	*probable novel intermediate*	*L. saintgironsiae* sp. nov. (sp. nov. inter 3)
ES4-C-A1	86.60	Intermediate	1.35	*probable novel intermediate*	*L. neocaledonica* sp. nov. (sp. nov inter 2)
ATI7-C-A4	86.62	Intermediate	1.48	*probable novel intermediate*	*L. haakeii* sp. nov. (sp. nov inter 4)
ATI7-C-A2	86.64	Intermediate	1.70	*probable novel intermediate*	*L. haakeii* sp. nov. (sp. nov inter 4)
FH2-C-A2	86.64	Intermediate	**2.07**	**possible novel intermediate**	*L. wolffii*
MCA1-C-A1	86.7	Intermediate	1.79	*probable novel intermediate*	*L. hartskeerlii* sp. nov. (sp. nov inter 5)
FH1-B-B1	86.82	Intermediate	0.52	*probable novel intermediate*	*L. perolatii* sp. nov. (sp. nov inter 1)
FH1-B-C1	86.95	Intermediate	0.94	*probable novel intermediate*	*L. perolatii* sp. nov. (sp. nov inter 1)

*MALDI-ToF identification scores ≥2.3 (in red) reliably identify the species. Scores in the uncertainty range 2.00–2.29 (bold) do not provide definitive identification. Color shade in T_m_ values indicates the clustering by increasing Tm values*.

### Whole-genome sequencing and phylogenetic analysis

The methods for DNA extraction, Whole-genome sequencing, assembly and species identification have been described in an earlier study (Thibeaux et al., 2018). Briefly, genomic DNA was prepared by collection of cells by centrifugation from an exponential-phase liquid culture and extraction with MagNA Pure 96 Instrument (Roche Life Science, Auckland, New Zealand). Next-generation sequencing was performed by the Mutualized Platform for Microbiology (P2M) at Institut Pasteur, using the Nextera XT DNA Library Preparation kit (Illumina), the NextSeq 500 sequencing systems (Illumina), and the CLC Genomics Workbench 9 software (Qiagen) for analysis. The whole genome sequences were studied as described previously and novel species were identified by pairwise comparisons of overall genomic relatedness indices ANI and AAI (Thibeaux et al., 2018).

### Ability of routine diagnostic PCR to detect the pathogenic species

The biological diagnosis of leptospirosis increasingly uses real time PCR. Here, we tested both a Taq-Man based PCR targeting *lipL32* for detection (Stoddard et al., 2009) and a *lfb1* SYBR Green I real time PCR that is used for both detection and genotyping (Merien et al., 2005; Perez and Goarant, 2010). Both techniques are used in a number of diagnostic or reference laboratories globally. The *lfb1* and *lipL32* sequences were retrieved from whole-genomes by blastn (Altschul et al., 1990), aligned and compared to the primers and probe described before (Supplementary Figure 1). Degenerate primers were designed to evaluate a possible improved detection of *lipL32* from all pathogens: lipL32-47Fd (GCATTACMGCTTGTGGTG) and lipL32-301Rd (CCGATTTCGCCWGTTGG) were used together with lipL32-189P (Stoddard et al., 2009) using the same PCR cycles. Using both qPCR designs, 0.2 ng pure DNA were amplified in a LightCycler 480 (*lipL32*) or in a LightCycler 2.0 (*lfb1*). Ct values were compared with the Ct values obtained from the same concentrations of pure DNA for each species with the original and the degenerate primers.

### Scanning electron microscopy imaging and morphology

Glass coverslips (12 × 12 mm, Menzel-Glaser) were placed into 24-well polystyrene plates (Corning) with 1 mL of a bacterial suspension at 10^6^ bacteria / mL and were incubated for 2 days. Coverslips were removed and rinsed once in PBS, then fixed in 4% paraformaldehyde / 1% glutaraldehyde for 15 min. Coverslips were rinse again in PBS and stained with 1% osmium tetroxide (OsO_4_ in PBS, Acros Organics) for 1 h. After staining, samples were dehydrated through a series of ethanol concentrations (25, 50, 70, 90, and 100% for 10 min each) before being desiccated using hexamethyldisilazane (HMDS, Acros Organics). Before visualization, samples were submitted to carbon sputtering to increase their electrical conductance (Em ACE600, Carbon Coater, Leica) and examined under a Jeol JSM IT-300 IntouchScope microscope (SED detector, 10 kV). Length, width and helix pitch were measured on 6–10 representative isolated cells to calculate mean dimensions.

## Results

### Environmental strain isolation

The major difficulty for isolating environmental leptospires resides in the ability to recover a clonal pure *Leptospira* culture from a complex polymicrobial arrangement. This procedure has been greatly facilitated by using the STAFF combination of antimicrobial agents (Chakraborty et al., 2011) which allows selective isolation of leptospires from contaminated samples. Earlier work has used quite similar combinations of agents to grow leptospires selectively with some success (Cousineau and McKiel, 1961; Jones et al., 1981). Although very effective, this is not sufficient to recover pure cultures as multiple *Leptospira* species can be present alongside in the initial sample. An additional step of clonal isolation from an individual isolated colony on an agar plate was necessary to recover clonal *Leptospira* cultures. We applied this procedure to attempt *Leptospira* isolation in 27 soil samples collected from 7 different sites throughout the mainland of New Caledonia (Thibeaux et al., 2018). After 5–10 days of liquid culture with STAFF, 23 cultures displayed the growth of *Leptospira*-like motile helical-shaped bacteria by dark-field microscopy. Positive liquid cultures were plated on solid agar plates and 26 individual colonies were sub-cloned in liquid EMJH according to their size, color and opacity (Figure 1).

### 16S rRNA *T_*m*_* analysis for rapid identification of *leptospira* at the clade-level

**Figure 2:**
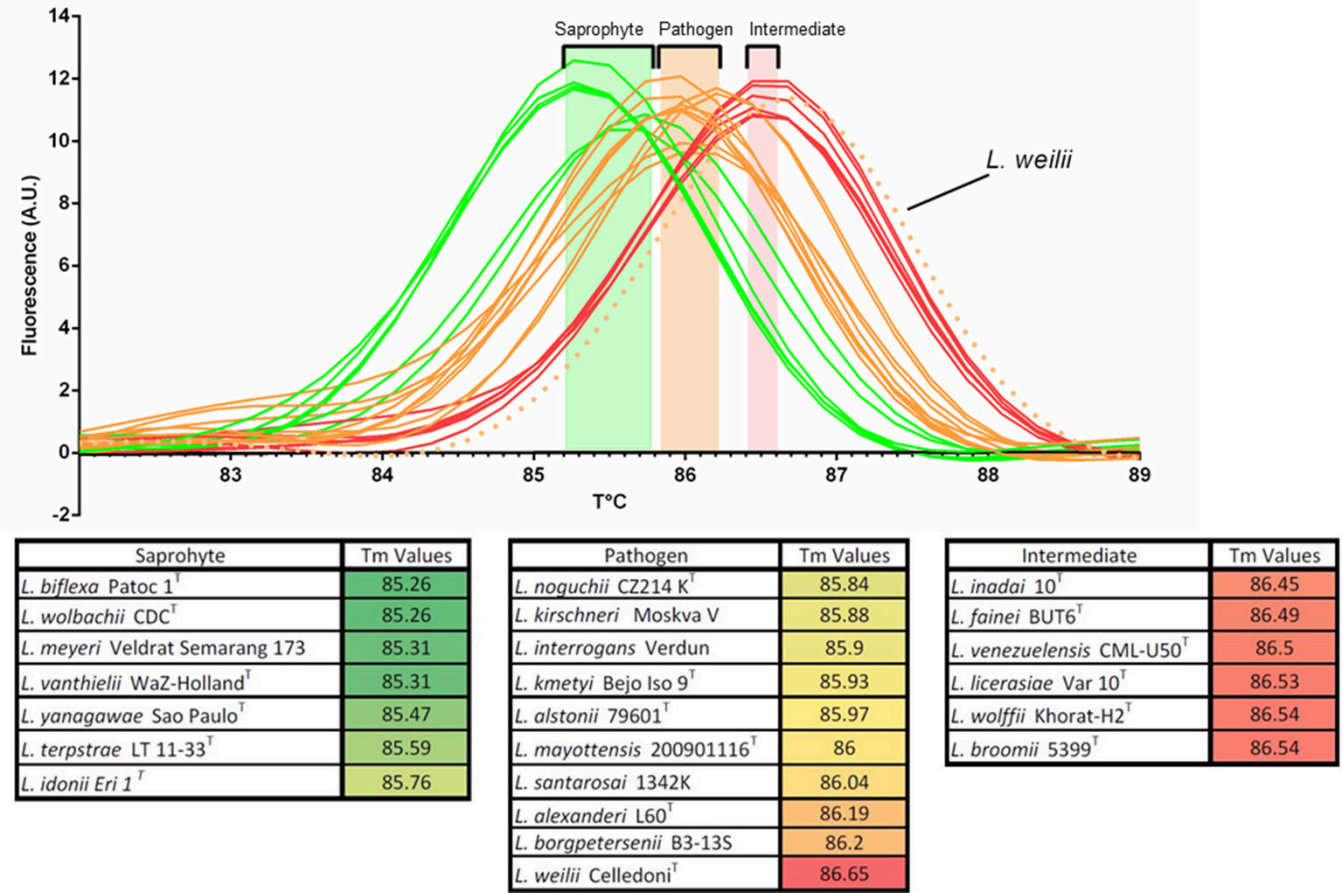
Melting peaks and melting temperatures (*T*_*m*_) of a 16S rRNA PCR product for representative strains from all 23 validly described *Leptospira* species, evidencing the clustering of Tm values with the notable exception of *Leptospira weilii*.

The melting temperature analysis of the amplicon of a region of the 16S rRNA gene (Mérien et al., 1992) conserved in the entire *Leptospira* genus allowed a rapid putative identification of the clade. Melting temperature values ranged from 85.26 to 86.65 and could separate the three clades within the *Leptospira* genus. *T*_*m*_ values of the saprophytic species were the lowest, ranging from 85.26 to 85.76 without overlapping with *T*_*m*_ values of the other species. Intermediate species had the highest melting temperature (86.45–86.54) while pathogenic species had *T*_*m*_ values ranging from 85.84 to 86.20, with the remarkable exception of *Leptospira weilii*, belonging to the pathogenic cluster but had the highest *T*_*m*_ value: 86.65°C, making this particular species the only outlier reported in this study (Figure 2.). Using these cluster-specific *T*_*m*_ ranges, we presumptively identified 10 saprophytic, 9 intermediate and 7 pathogenic *Leptospira* strains from pure cultures (Table 1, Step I).

### MALDI-ToF analysis for rapid identification of *leptospira* species

Using the imported MS database on our MALDI-ToF apparatus with our strain collection, culture medium and preparation reagents, we correctly identified all 27 reference strains at the species level with identification scores ≥2.30 and not significantly different from scores obtained with our in-house database (data not shown). These results confirm the portability of MS databases. MALDI-ToF MS was then used to attempt identification of our 26 isolates. Pairwise comparison with reference spectra representative of the 23 known *Leptospira* species was performed to identify isolates at the species level. Referring to the most complete database of reference spectra is a prerequisite to make use of the MALDI-ToF as an identification strategy of unknown species. In this case, an identification that fail to be matched with reference species MS with a valid score ≥2.3 would possibly point to a novel species deserving WGS analysis. Applying this identification strategy to our environmental isolates, 5 isolates had a high-confidence identification score (≥2.3) and were identified as *L. kmetyi* (*n* = 2), and *L. meyeri* (*n* = 3) (Table 1, Step II). The other 21 isolates failed to be matched with known species, suggesting possible novel species. Interestingly, we noticed for these isolates that even though the score was below the identification threshold, the proposed closest hit was always in good agreement with the proposed clade putatively assigned with the *T*_*m*_ of the 16S rRNA PCR product (Table 1, Step II).

**Figure 3:**
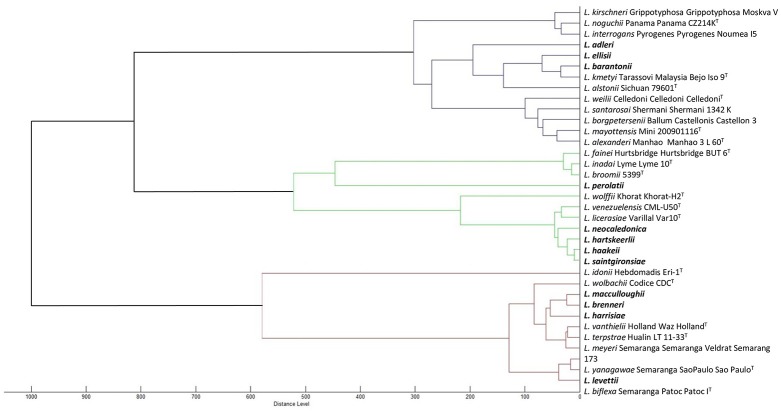
Dendrogram based on the comparison of MALDI-ToF mass spectra of the 35 *Leptospira* species. Novel species are indicated in bold.

Furthermore, we have refined the identification of these 21 unidentified cultures by performing pairwise comparison with each other to identify isolates belonging to the same species, if any. Some cultures were identified as identical putative novel species (comparison score ≥2.3), pointing to 13 possible or putative novel species (Table 1 and Figure 3).

### Whole-genome sequencing

As the last step of the stepwise identification, the whole-genome sequence of all 26 isolates were determined. The 5 isolates identified by MALDI-ToF MS as *L. meyeri* (*n* = 3) or *L. kmetyi* (*n* = 2) were confirmed members of these species by ad-hoc analysis of whole-genome sequences (Thibeaux et al., 2018). Of 21 unknown isolates possibly representing novel species, one isolate (FH2-C-A2), assigned to a possible novel intermediate species, was formally identified as *L. wolffii*. Of note, the MALDI-ToF identification score with *L. wolffii* Khorat-H2^T^ was 2.07, in the range of the species uncertainty suggested in a former study (Rettinger et al., 2012) and recommended by the manufacturer. In addition, 20 isolates were confirmed to belong to 12 novel *Leptospira* species (3 pathogens, 5 intermediates and 4 saprophytes) by WGS and genome analysis (Thibeaux et al., 2018) (Table 1, step III).

### Description of novel species

**Figure 4:**
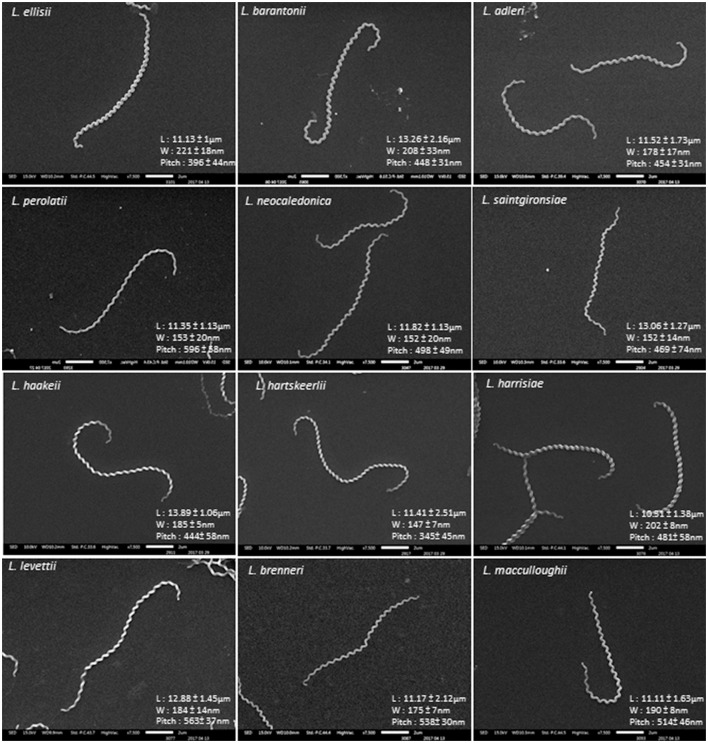
SEM morphology of representative cells of novel *Leptospira* species. L, length; W, width.

Figure 4 shows the morphology of novel species obtained by scanning electron microscopy. The position of novel species in a 16S rRNA phylogenetic tree is shown in Supplementary Figure 2. We propose the following names and descriptions for the novel species:

#### Description of *Leptospira ellisii* sp. nov

*Leptospira ellisii* (e.li'si.i N.L gen. masc. n. *ellisii* of Ellis, named after Dr William A. Ellis, an Irish veterinary microbiologist who made significant contributions to the study of *Leptospira* and leptospirosis in animals). Morphology observed under scanning electron microscopy is consistent with those for the genus. Cells have helical morphology, finely thin and usually curved at each end, forming a semicircular hook. Cells are 11.13 ± 1 μm long and 221 ± 18 nm in diameter with a pitch of 396 ± 44 nm. Motile and aerobic. Cells grow well in liquid and solid EMJH medium (1.2% agar) at 30°C. Growth in the presence of antibiotics, such as 5-fluorouracil, 1 mg/mL; Amphotericin B, 50 μg/mL; Sulfamethoxazole, 400 μg/mL; Trimethoprim, 200 μg/mL and Fosmomycin, 4 mg/mL was also observed. The genomic G+C content of the type strain is 47.8%. This strain could neither induce signs or symptoms of infection in the hamster model nor be recovered from hamster blood. Similarly, it could not be detected in mouse urine or kidney after inoculation. The type strain isolated to date is ATI7-C-A5^T^, isolated from a soil sample in the South Province of New Caledonia. Genome Accession Number is NPEF00000000. Belonging to the cluster of “pathogens,” this species was formerly described as *Leptospira* sp. nov. patho 1 (Thibeaux et al., 2018).

#### Description of *Leptospira barantonii* sp. nov

*Leptospira barantonii* (ba.ran.to'ni.i. N.L gen. masc. n. *barantonii* of Baranton, named after Pr Guy Baranton, a French microbiologist who made significant contributions to the study of Spirochaetes). Morphology observed under scanning electron microscopy is consistent with those for the genus. Cells have helical morphology, finely thin and usually curved at each end, forming a semicircular hook. Cells are 13.26 ± 2.16 μm long and 208 ± 33 nm in diameter with a pitch of 448 ± 31 nm. Motile and aerobic. Cells grow well in liquid and solid EMJH medium (1.2% agar) at 30°C. Growth in the presence of antibiotics, such as 5-fluorouracil, 1 mg/mL; Amphotericin B, 50 μg/mL; Sulfamethoxazole, 400 μg/mL; Trimethoprim, 200 μg/mL and Fosmomycin, 4 mg/mL was also observed. The genomic G+C content of the type strain is 43.96%. This strain could neither induce signs or symptoms of infection in the hamster model nor be recovered from hamster blood. Similarly, it could not be detected in mouse urine or kidney after inoculation. The type and only strain isolated to date is FH4-C-A1^T^, isolated from a soil sample in the North Province of New Caledonia. Genome Accession Number is NPDS00000000. Belonging to the cluster of “pathogens,” this species was formerly described as *Leptospira* sp. nov. patho 2 (Thibeaux et al., 2018).

#### Description of *Leptospira adleri* sp. nov

*Leptospira adler* (a.dle'ri. N.L gen. masc. n. *adleri* of Adler, named after Pr Ben Adler, an Australian microbiologist who made significant research contributions in many aspects of *Leptospira* and leptospirosis). Morphology observed under scanning electron microscopy is consistent with those for the genus. Cells have helical morphology, finely thin and usually curved at each end, forming a semicircular hook. Cells are 11.52 ± 1.73 μm long and 178 ± 17 nm in diameter with a pitch of 454 ± 31 nm. Motile and aerobic. Cells grow well in liquid and solid EMJH medium (1.2% agar) at 30°C. Growth in the presence of antibiotics, such as 5-fluorouracil, 1 mg/mL; Amphotericin B, 50 μg/mL; Sulfamethoxazole, 400 μg/mL; Trimethoprim, 200 μg/mL and Fosmomycin, 4 mg/mL was also observed. The genomic G+C content of the type strain is 43.55%. This strain could neither induce signs or symptoms of infection in the hamster model nor be recovered from hamster blood. Similarly, it could not be detected in mouse urine or kidney after inoculation. The type strain isolated to date is FH2-B-D1^T^, isolated from a soil sample in the North Province of New Caledonia. Genome Accession Number is NPDU00000000. Belonging to the cluster of “pathogens,” this species was formerly described as *Leptospira* sp. nov. patho 3 (Thibeaux et al., 2018).

#### Description of *Leptospira perolatii* sp. nov

*Leptospira perolatii* (pe.ro.la'ti.i. N.L gen. masc. n. *perolatii* of Perolat, named after Dr Philippe Perolat, a French microbiologist who made significant contributions to the study of *Leptospira*). Morphology observed under scanning electron microscopy is consistent with those for the genus. Cells have helical morphology, finely thin and usually curved at each end, forming a semicircular hook but spiralstructure of this isolate appear more relaxed. Cells are 11.35 ± 1.13 μm long and 153 ± 20 nm in diameter with a pitch of 596 ± 58 nm. Motile and aerobic. Cells grow well in liquid and solid EMJH medium (1.2% agar) at 30°C. Growth in the presence of antibiotics, such as 5-fluorouracil, 1 mg/mL; Amphotericin B, 50 μg/mL; Sulfamethoxazole, 400 μg/mL; Trimethoprim, 200 μg/mL and Fosmomycin, 4 mg/mL was also observed. The genomic G+C content of the type strain is 42.36%. The type strain isolated to date is FH1-B-B1^T^, isolated from a soil sample in the North Province of New Caledonia. Genome Accession Number is NPDZ00000000. Belonging to the cluster of “intermediates,” this species was formerly described as *Leptospira* sp. nov. inter 1 (Thibeaux et al., 2018).

#### Description of *Leptospira neocaledonica* sp. nov

*Leptospira neocaledonica* (neo.ca.le.do.ni'ca. N.L gen. fem. adj. *neocaledonica* reflecting its geographic origin in New Caledonia). Morphology observed under scanning electron microscopy is consistent with those for the genus. Cells have helical morphology, finely thin and usually curved at each end, forming a semicircular hook. Cells are 11.82 ± 1.13 μm long and 152 ± 20 nm in diameter with a pitch of 498 ± 49 nm. Motile and aerobic. Cells grow well in liquid and solid EMJH medium (1.2% agar) at 30°C. Growth in the presence of antibiotics, such as 5-fluorouracil, 1 mg/mL; Amphotericin B, 50 μg/mL; Sulfamethoxazole, 400 μg/mL; Trimethoprim, 200 μg/mL and Fosmomycin, 4 mg/mL was also observed. The genomic G+C content of the type strain is 40.17%. The type and only strain isolated to date is ES4-C-A1^T^, isolated from a soil sample in the North Province of New Caledonia. Genome Accession Number is NPEA00000000. Belonging to the cluster of “intermediates,” this species was formerly described as *Leptospira* sp. nov. inter 2 (Thibeaux et al., 2018).

#### Description of *Leptospira saintgironsiae* sp. nov

*Leptospira saintgironsiae* (saint.gi.ron'si.ae. N.L gen. fem. n. *saintgironsiae* of Saint-Girons, named after Dr Isabelle Saint-Girons, a French microbiologist who made significant contributions to the study of Spirochaetes). Morphology observed under scanning electron microscopy is consistent with those for the genus. Cells have helical morphology, finely thin and usually curved at each end, forming a semicircular hook. Cells are 13.06 ± 1.27 μm long and 152 ± 14 nm in diameter with a pitch of 469 ± 74 nm. Motile and aerobic. Cells grow well in liquid and solid EMJH medium (1.2% agar) at 30°C. Growth in the presence of antibiotics, such as 5-fluorouracil, 1 mg/mL; Amphotericin B, 50 μg/mL; Sulfamethoxazole, 400 μg/mL; Trimethoprim, 200 μg/mL and Fosmomycin, 4 mg/mL was also observed. The genomic G+C content of the type strain is 45.82%. The type and only strain isolated to date is FH4-C-A2^T^, isolated from a soil sample in the North Province of New Caledonia. Genome Accession Number is NPDR00000000. Belonging to the cluster of “intermediates,” this species was formerly described as *Leptospira* sp. nov. inter 3 (Thibeaux et al., 2018).

#### Description of *Leptospira haakeii* sp. nov

*Leptospira haakeii* (ha.a'kei.i. N.L gen. masc. n. *haakeii* of Haake, named after Pr David Haake, an American microbiologist who made significant contributions to the study of *Leptospira*, notably on the role of outer membrane proteins in the pathophysiology of leptospirosis*)*. Morphology observed under scanning electron microscopy is consistent with those for the genus. Cells have helical morphology, finely thin and usually curved at each end, forming a semicircular hook. Cells are 13.89 ± 1.06 μm long and 183 ± 5 nm in diameter with a pitch of 444 ± 58 nm. Motile and aerobic. Cells grow well in liquid and solid EMJH medium (1.2% agar) at 30°C. Growth in the presence of antibiotics, such as 5-fluorouracil, 1 mg/mL; Amphotericin B, 50 μg/mL; Sulfamethoxazole, 400 μg/mL; Trimethoprim, 200 μg/mL and Fosmomycin, 4 mg/mL was also observed. The genomic G+C content of the type strain is 39.8%.The type strain isolated to date is ATI7-C-A4^T^, isolated from a soil sample in the South Province of New Caledonia. Genome Accession Number is NPEG00000000. Belonging to the cluster of “intermediates,” this species was formerly described as *Leptospira* sp. nov. inter 4 (Thibeaux et al., 2018).

#### Description of *Leptospira hartskeerlii* sp. nov

*Leptospira hartskeerlii* (hart.skeer'li.i. N.L gen. masc. n. *hartskeerlii* of Harskeerl named after Dr Rudy Harskeerl, an Dutch microbiologist who made significant contributions to the study of all aspects of leptospirosis, notably diagnosis and epidemiology). Morphology observed under scanning electron microscopy is consistent with those for the genus. Cells have helical morphology, finely thin and usually curved at each end, forming a semicircular hook. Cells are 11.41 ± 2.51 μm long and 147 ± 7 nm in diameter with a pitch of 345 ± 45 nm. Motile and aerobic. Cells grow well in liquid and solid EMJH medium (1.2% agar) at 30°C. Growth in the presence of antibiotics, such as 5-fluorouracil, 1 mg/mL; Amphotericin B, 50 μg/mL; Sulfamethoxazole, 400 μg/mL; Trimethoprim, 200 μg/mL and Fosmomycin, 4 mg/mL was also observed. The genomic G+C content of the type strain is 40.47%. The type strain isolated to date is MCA2-B-A3^T^, isolated from a soil sample in the North Province of New Caledonia. Genome Accession Number is NPDL00000000. Belonging to the cluster of “intermediates,” this species was formerly described as *Leptospira* sp. nov. inter 5 (Thibeaux et al., 2018).

#### Description of *Leptospira harrisiae* sp. nov

*Leptospira harrisiae* (ha.rri'si.ae. N.L gen. fem. n. *harrisiae* of Harris named after Dr Virginia G. Harris, an American microbiologist who made significant contributions to the study of nutrition and physiology of *Leptospira*). Morphology observed under scanning electron microscopy is consistent with those for the genus. Cells have helical morphology, finely thin and usually curved at each end, forming a semicircular hook. Cells are 10.31 ± 1.38 μm long and 202 ± 8 nm in diameter with a pitch of 481 ± 58 nm. Motile and aerobic. Cells grow well in liquid and solid EMJH medium (1.2% agar) at 30°C. Growth in the presence of antibiotics, such as 5-fluorouracil, 1 mg/mL; Amphotericin B, 50 μg/mL; Sulfamethoxazole, 400 μg/mL; Trimethoprim, 200 μg/mL and Fosmomycin, 4 mg/mL was also observed. The genomic G+C content of the type strain is 37.86%. The type strain isolated to date is FH2-B-A1^T^, isolated from a soil sample in the North Province of New Caledonia. Genome Accession Number is NPDX00000000. Belonging to the cluster of “saprophytes,” this species was formerly described as *Leptospira* sp. nov. sapro 1 (Thibeaux et al., 2018).

#### Description of *Leptospira levettii* sp. nov

*Leptospira levettii* (le'vet.ti.i. N.L gen. masc. n. *levettii* of Levett named after Dr Paul N. Levett, a Canadian microbiologist who made significant contributions to the study of *Leptospira* and leptospirosis). Morphology observed under scanning electron microscopy is consistent with those for the genus. Cells have helical morphology, finely thin and usually curved at each end, forming a semicircular hook. Cells are 12.88 ± 1.45 μm long and 184 ± 14 nm in diameter with a pitch of 563 ± 14 nm. Motile and aerobic. Cells grow well in liquid and solid EMJH medium (1.2% agar) at 30°C. Growth in the presence of antibiotics, such as 5-fluorouracil, 1 mg/mL; Amphotericin B, 50 μg/mL; Sulfamethoxazole, 400 μg/mL; Trimethoprim, 200 μg/mL and Fosmomycin, 4 mg/mL was also observed. The genomic G+C content of the type strain is 37.61%. The type strain isolated to date is MCA2-B-A1^T^, isolated from a soil sample in the North Province of New Caledonia. Genome Accession Number is NPDM00000000. Belonging to the cluster of “saprophytes,” this species was formerly described as *Leptospira* sp. nov. sapro 2 (Thibeaux et al., 2018).

#### Description of *Leptospira brenneri* sp. nov

*Leptospira brenneri* (bre.nne.ri. N.L gen. masc. n. *brenneri* of Brenner named after Dr Don J. Brenner, an American microbiologist who made significant contributions to the study of *Leptospira*, notably pioneered the field of molecular taxonomy). Morphology observed under scanning electron microscopy is consistent with those for the genus. Cells have helical morphology, finely thin and usually curved at each end, forming a semicircular hook. Cells are 11.17 ± 2.12 μm long and 175 ± 7 nm in diameter with a pitch of 538 ± 30 nm. Motile and aerobic. Cells grow well in liquid and solid EMJH medium (1.2% agar) at 30°C. Growth in the presence of antibiotics, such as 5-fluorouracil, 1 mg/mL; Amphotericin B, 50 μg/mL; Sulfamethoxazole, 400 μg/mL; Trimethoprim, 200 μg/mL and Fosmomycin, 4 mg/mL was also observed. The genomic G+C content of the type strain is 38.34%. The type and only strain isolated to date is JW2-C-A2^T^, isolated from a soil sample in the North Province of New Caledonia. Genome Accession Number is NPDQ00000000. Belonging to the cluster of “saprophytes,” this species was formerly described as *Leptospira* sp. nov. sapro 3 (Thibeaux et al., 2018).

#### Description of *Leptospira macculloughii* sp. nov

*Leptospira macculloughii* (mac.cu.llou'ghi.i. N.L gen. masc. n. *macculloughii* of McCullough named after Dr. Willard G. McCullough, an American microbiologist who made significant contributions to the study of the nutritional requirements of *Leptospira*). Morphology observed under scanning electron microscopy is consistent with those for the genus. Cells have helical morphology, finely thin and usually curved at each end, forming a semicircular hook. Cells are 11.11 ± 1.63 μm long and 190 ± 8 nm in diameter with a pitch of 514 ± 46 nm. Motile and aerobic. Cells grow well in liquid and solid EMJH medium (1.2% agar) at 30 °C. Growth in the presence of antibiotics, such as 5-fluorouracil, 1 mg/mL; Amphotericin B, 50 μg/mL; Sulfamethoxazole, 400 μg/mL; Trimethoprim, 200 μg/mL and Fosmomycin, 4 mg/mL was also observed. The genomic G+C content of the type strain is 37.93%. The type and only strain isolated to date is ATI2-C-A1^T^, isolated from a soil sample in the North Province of New Caledonia. Genome Accession Number is NPEK00000000. Belonging to the cluster of “saprophytes,” this species was formerly described as *Leptospira* sp. nov. sapro 4 (Thibeaux et al., 2018).

### Evaluation and optimization of qPCR targeting the pathogenic species

**Figure 5:**
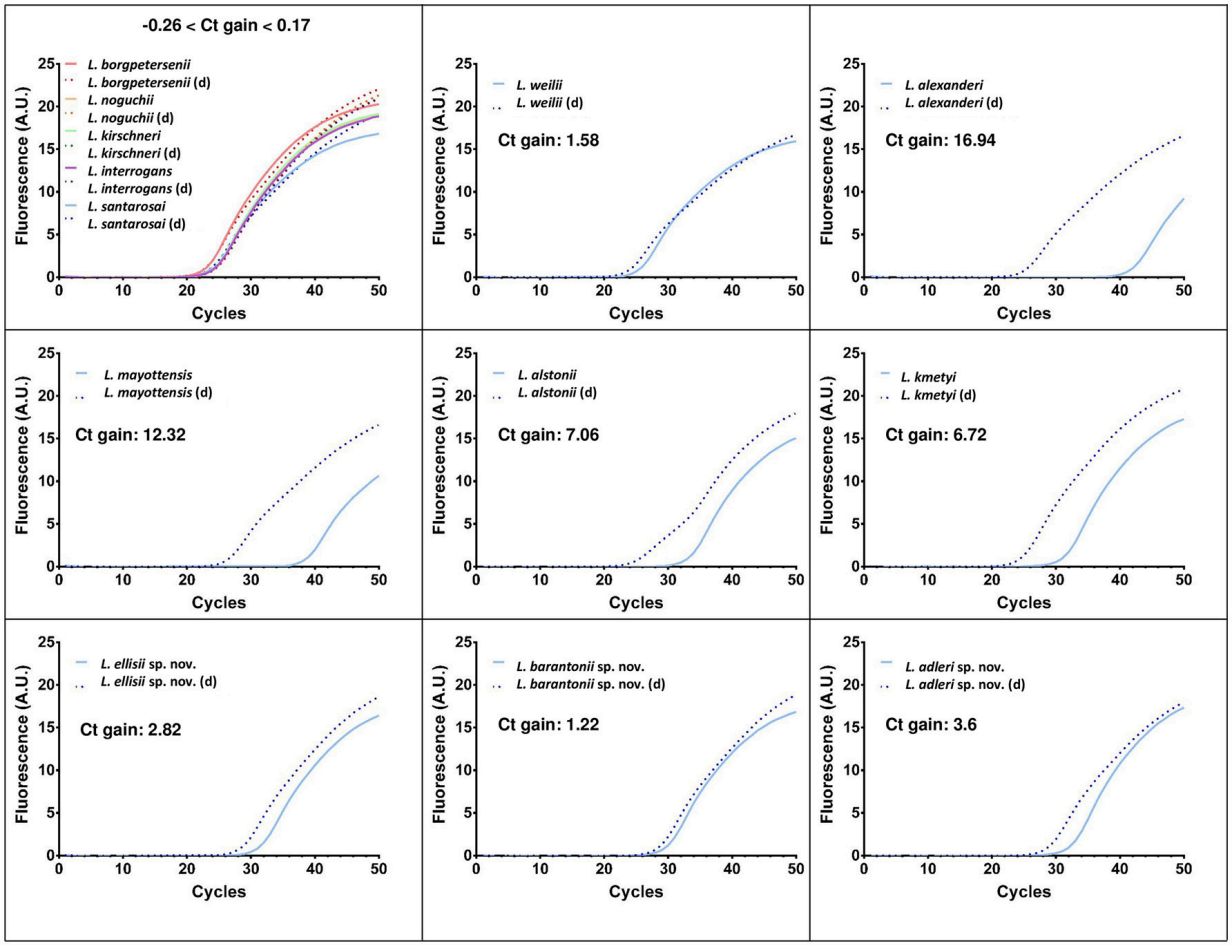
Real-time PCR amplification curves and Ct gains using the original (Merien et al., 2005) and a modified qPCR using degenerate primers (d) described in this paper targeting *lipL32* to detect pathogenic *Leptospira*.

Alignments of *lipL32* and *lfb1* gene sequences from previously described and novel pathogenic species revealed polymorphisms resulting in mismatches in the primers and probe of the diagnostic qPCR designs tested. Using routine protocols, the novel species were still amplified and detected, but with much higher Cycle threshold (Ct) values, pointing to a low sensitivity of these diagnostic PCR for these novel species as well as others. Figure 5 shows the real-time PCR amplification curves and Ct values for qPCR using *lipL32* as the target. The degenerate *lipL32* primers lipL32-47Fd and lipL32-301Rd (Supplementary Figure 1) used in conjunction with the probe lipL32-189P described in the original design (Stoddard et al., 2009) demonstrated a higher efficacy to detect all species from the cluster “pathogens,” Indeed, using these degenerate *lipL32* primers, an astonishing Ct gain (showing improved sensitivity) of 16.94 cycles was observed for *L. alexanderi*. PCR sensitivity was also greatly increased for *L. mayottensis* (a 12.34-cycle gain), *L. alstonii* (7.06 cycles), *L. kmetyi* (6.72 cycles), *L. adleri* sp. nov. (3.6 cycles), *L. ellisii* sp. nov. (1.82 cycles), *L. weilii* (1.58 cycles) and *L. barantonii* sp. nov. (1.22 cycles). For *L. borgpetersenii, L. noguchii, L. kirschneri, L. interrogans* and *L. santarosai*, PCR sensitivity was barely affected with respectively + 0.17 cycle, −0.26 cycle, −0.08 cycle, −0.17 cycle, +0.16 cycle. All these results are summarized in Supplementary Table 2. A proper evaluation of this new qPCR design in terms of sensitivity and specificity would still be needed before it could be used for diagnostic purpose.

Concerning Ct values and amplification curves for qPCR using *lfb1* as a target, important discrepancies were also observed with Ct values ranging from 23.43 to 47.48 also resulting from important sequence polymorphisms affecting primer match. Comparative Ct values and Melting temperatures obtained with the *lfb1* qPCR (Merien et al., 2005) are presented in Supplementary Table 3.

## Discussion

In the last decade, the size of the genus *Leptospira* has increased through the description of novel species from clinical specimens, either belonging to the intermediate cluster like *Leptospira broomii* (Levett et al., 2006), *Leptospira licerasiae* (Matthias et al., 2008), *Leptospira wolffii* (Slack et al., 2008), *Leptospira venezuelensis* (Puche et al., 2018) or to the pathogenic cluster with *Leptospira mayottensis* (Bourhy et al., 2014) and novel species from soils (Thibeaux et al., 2018). Simultaneously, concern is increasing about intermediate species being etiological agents of human disease (Tsuboi et al., 2017). This highlights both the need to continue *Leptospira* isolation to better evaluate the diversity of this genus (Girault et al., 2017) and a growing need for a universal PCR assay for the diagnostic of both intermediate and pathogenic *Leptospira* from clinical specimens.

In this study, we first report the stepwise identification of 26 *Leptospira* isolates from tropical freshwater soils in New Caledonia. Studying *Leptospira* in water and soil has first revealed (Ganoza et al., 2006; Viau and Boehm, 2011), then confirmed an overlooked biodiversity within the genus *Leptospira* (Thibeaux et al., 2018). These studies support the concept that leptospires are primarily telluric saprophytic bacteria and that only some members have evolved toward pathogenicity. From an epidemiological viewpoint, this illustrates the importance of soils in the infection cycle by virulent strains, pointing to the notion that ecosystems that support the survival of virulent leptospires should be considered as a passive, yet major reservoir in the epidemiology of the disease. Not only water content and pH, but a number of soil parameters may determine the suitability of a soil to leptospires and condition the risk to human and animal health.

To progress in this understanding, the discovery of this high biodiversity also reinforces the need of accurate isolation and identification techniques as well as relevant diagnostic tools. For isolation, an essential technical step was to plate positive liquid cultures on EMJH-agar to pick each isolate from a single colony, as shown by the diversity on plates from a single liquid culture (Figure 1). For identification, we used a polyphasic approach based on three sequential steps: (i) melting temperature analysis of a PCR product within the 16S rRNA gene, (ii) MALDI-ToF MS pairwise comparisons to a broad MS database and (iii) WGS analyses.

The recovery of isolates from the cluster “pathogens” from the environment is fastidious and challenging (Henry et al., 1971). Pathogenic leptospires have been historically isolated from soils in Malaysia (Baker and Baker, 1970; Alexander et al., 1975), using inoculation to a large number of susceptible animals, a technique nowadays ethically unacceptable. In addition, this animal-based strategy is biased since only strains leading to hamster disease could be recovered. Only virulent strains at a concentration above the infective dose can be recovered with this technique. Attempts to isolate *Leptospira* from the environment has led to the description of *L. kmetyi* from soil and other successful isolation of pathogenic leptospires from the environment in Malaysia (Slack et al., 2009; Ridzlan et al., 2010; Benacer et al., 2013). The use of selective agents to isolate leptospires from complex samples was imagined in the early 1960s (Cousineau and McKiel, 1961), successfully used later (Jones et al., 1981), and a revised combination was successfully used recently (Chakraborty et al., 2011).

Comparative analysis of the small ribosomal 16S rRNA subunit gene has been regarded as a standard for bacterial species identification for decades. In *Leptospira*, this gene was also largely used as a target for diagnostics (Mérien et al., 1992; Smythe et al., 2002) and typing (Ahmed et al., 2006; Guernier et al., 2018). Sequences of partial 16S rRNA gene are also widely used to identify *Leptospira* at the species level, either by direct sequencing (Benacer et al., 2013; Saito et al., 2014; Chaiwattanarungruengpaisan et al., 2018) or using High Resolution Melting (Pelaez Sanchez et al., 2017). However, partial sequences of the 16S rRNA gene might not be able to differentiate between closely-related species (Guernier et al., 2018), possibly leading to inconclusive or even improper identification at the species level. In recent studies, many isolates were putatively identified as *L. alstonii, L. wolffii*, or *L. licerasiae* based on their 16S rRNA gene sequence (Chakraborty et al., 2011; Benacer et al., 2013; Saito et al., 2013; Azali et al., 2016). Here, we suggest that these isolates would deserve further characterization. Hence, we propose that the simple melting temperature after real time PCR of a short portion of the 16S rRNA gene can presumptively classify an isolate in one of the 3 *Leptospira* clusters (pathogens, intermediates and saprophytes), with the notable exception of *L. weilii* (Table 1 and Figure 2).

We then used MALDI-ToF to identify pure cultures by creating a broad MS database allowing automated pairwise comparison to all validly described species. This technology is emerging in the field of medical microbiology and already used in the genus *Leptospira* (Djelouadji et al., 2012; Rettinger et al., 2012; Calderaro et al., 2014). As expected from previous work, we were able to identify some environmental isolates as members of species already described in all three *Leptospira* clusters. The MSP dendrogram deduced from the MALDI-ToF MS fingerprints clearly separated the three main clusters within the genus *Leptospira* and successfully distinguished all previously described species (Figure 3). Lastly, we used whole-genome sequence analyses to provide an identification of species (Thibeaux et al., 2018). This unambiguous identification of species confirmed the validity of the MALDI-ToF identifications, with correct species assignment for identification scores ≥2.3. WGS analyses also identified one isolate as *L. wolffii*; pairwise comparisons of MS to *L. wolffii* Khorat KH2^T^ had an identification score of 2.07, in the “uncertain identification” range. Within this range, all other isolates corresponded to novel species not included in the MS database. Lastly, isolates with an identification score below 2.0 all corresponded to novel species. After WGS analyses and phylogenetic assignment of novel species, we confirmed the correct identification of the cluster based on 16S rRNA melting peaks (Figure 2). Compared to MALDI-ToF identifications, we also noted that the closest match was always a species from the same *Leptospira* cluster (Figure 3). Taken together, we propose a sequential approach to isolate and identify environmental *Leptospira*. Although MALDI-ToF bacterial identification is not readily available in every research laboratory, the possibility to halt the identification process by freezing the protein extract (see section Materials and Methods) offers the possibility to refer specimens to distant laboratories. MALDI-ToF-based species identification requires a broad MS database, which is provided for research use as part of our article (Supplementary: MspExport_BDD-UREL.btmsp file available online).

The diagnostic and surveillance of leptospirosis has mostly used serology and culture for decades. Since the advent of molecular techniques, PCR and real time PCR have been most widely used, frequently leading to abandon culture isolation (Perez and Goarant, 2010; Girault et al., 2017). However, most of the current molecular techniques specifically target the pathogenic cluster of *Leptospira*, or take *L. interrogans* sequences as the model target. The real time PCR primers (and eventually probe) may not be fitted to all relevant species. We tested two real time PCR assays widely used for diagnosis over the 10 validly described pathogenic species as well as the 3 novel pathogens. Surprisingly, we found that a TaqMan-based real time PCR targeting *lipL32* that is widely used (Stoddard et al., 2009) was poorly sensitive for the detection of several *Leptospira* from the pathogenic cluster, including *L. alexanderi* and *L. mayottensis* (Supplementary Table 2). Using a new set of degenerate primers with the original probe of a TaqMan assay used widely, we significantly improved the detection of these species (Figure 5 and Supplementary Table 2). Similarly, a SYBR-Green I technique targeting *lfb1* that is used for diagnostic purpose (Merien et al., 2005) was poorly sensitive for the detection of *L. alstonii, L. kmetyi* and the novel pathogenic species (Supplementary Table 3). This suggests that the high specificity of diagnostic PCR might lead to miss the detection of several rare or atypical *Leptospira* strains. The modified design proposed here should be tested further for validation of sensitivity and specificity, but may already be useful for exploratory studies.

LipL32 is known to be present in all species from both “pathogens” and “intermediate” clusters. However, this gene is not necessary for virulence (Murray et al., 2009) and is also present in low-virulence species described recently (Thibeaux et al., 2018). In addition and because of gene sequence polymorphisms, the most used real time PCR techniques targeting *lipL32* do not detect intermediate species (Levett et al., 2005; Stoddard et al., 2009). Genus-specific targets might be used in clinical specimens, where only virulent leptospires are expected to be present. However and because recent work strongly suggests very distinct virulence levels within the pathogenic cluster (Lehmann et al., 2014; Xu et al., 2016; Thibeaux et al., 2018), molecular detection in environmental samples should either be followed by typing (Thibeaux et al., 2017) or interpreted with caution until validated virulence-specific detection methods are available. The identification of relevant target genes by comparative genomics will help improve the relevance of future diagnostic tools.

## Author contributions

CG conceived and designed the study. CG, RT, DG, EB, and MESG designed isolation strategy and isolated leptospires. CG, RT, EB, and MESG developed and implemented molecular strategy. DG, AR, and CG developed MALDI-ToF techniques and constructed MSP database. GI, MP, and CG WGS and analysis. RT, AD, and MM did the Electron Microscopy. CG, RT, DG, GI, and MP analyzed the data. CG, RT, and DG wrote the manuscript. MP, GI, and AR revised and edited the manuscript. All authors validated the manuscript.

### Conflict of interest statement

The authors declare that the research was conducted in the absence of any commercial or financial relationships that could be construed as a potential conflict of interest.

## Supplementary material

MALDI-ToF database with mass spectra of 35 *Leptospira* species (btmsp file).
