# Supplementary material for: Biodiversity of Environmental Leptospira: Improving Identification and Revisiting the Diagnosis
Source: Front Microbiol. 2018 May 1;9:816. doi: 10.3389/fmicb.2018.00816 (PMC5938396; doi:10.3389/fmicb.2018.00816)
Supplement: Supplementary file 2 [file Image_1.PDF]

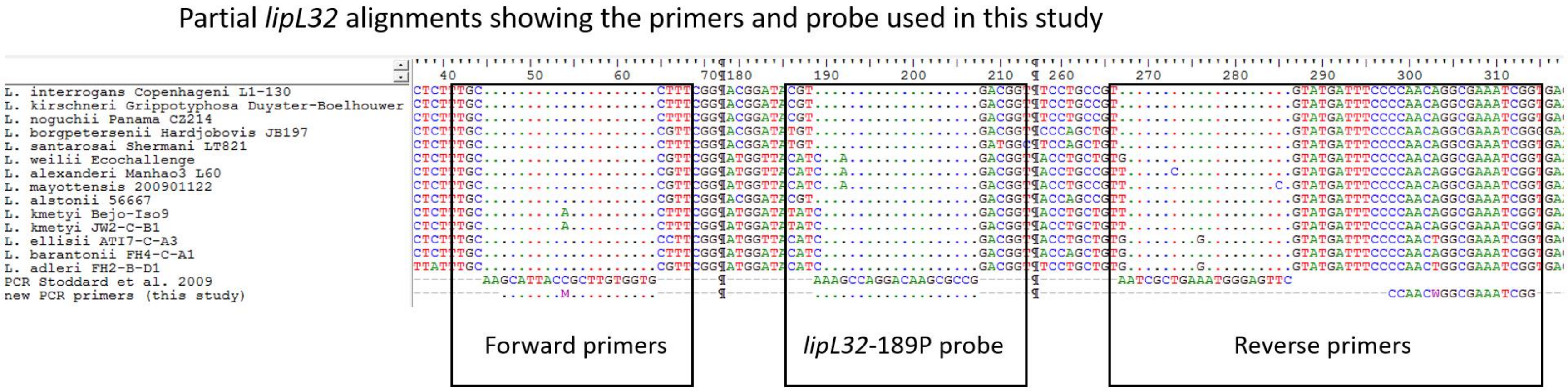

Supplementary Figure 1: *lipL32* alignments with standard and novel primers
