# Supplementary figures and images for: Biodiversity of Environmental Leptospira: Improving Identification and Revisiting the Diagnosis
Source: Front Microbiol. 2018 May 1;9:816. doi: 10.3389/fmicb.2018.00816 (PMC5938396; doi:10.3389/fmicb.2018.00816)

**Supplementary Figure 2:** 16S rRNA phylogeny of the *Leptospira* genus with novel species in color

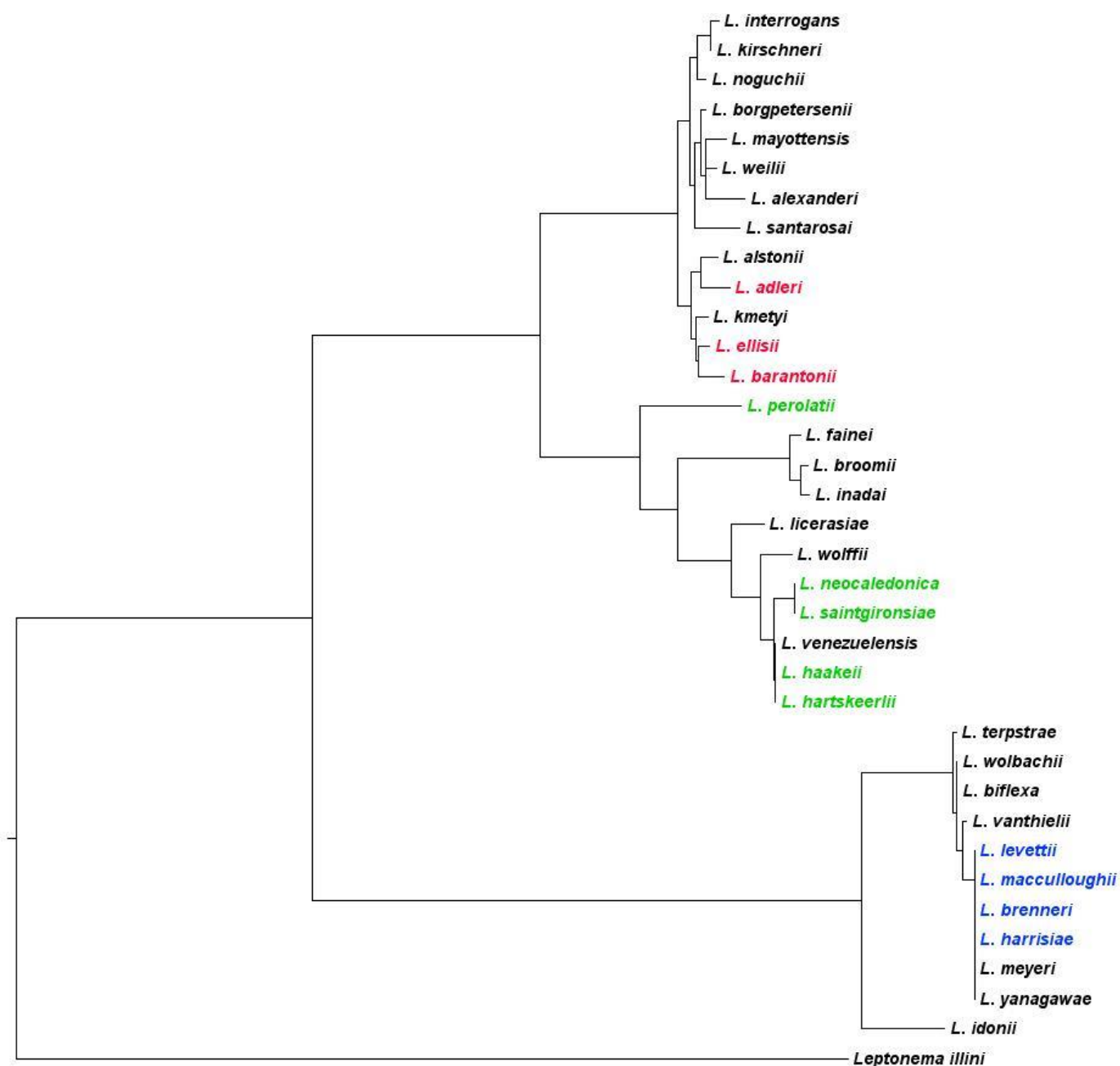

Supplement: Supplementary file 3 [file Image_2.pdf]
