# Supplementary material for: Biodiversity of Environmental Leptospira: Improving Identification and Revisiting the Diagnosis
Source: Front Microbiol. 2018 May 1;9:816. doi: 10.3389/fmicb.2018.00816 (PMC5938396; doi:10.3389/fmicb.2018.00816)
Supplement: Supplementary file 4 [file Table_1.PDF]

Supplementary Table I. *Leptospira* reference strains and isolates

Bold lines indicate strains used to confirm the portability of the MSP

Shaded lines indicate New Caledonian environmental isolates

N°	Clade	Specie	Serogroup	Serovar	Strain
1	**pathogenic**	***L. interrogans***	**Bataviae**	**Bataviae**	**Van Tienen**
2	**pathogenic**	***L. interrogans***	**Pomona**	**Pomona**	**Pomona**
3	**pathogenic**	***L. interrogans***	**Australis**	**Bratislava**	**Jetz Bratislava**
4	**pathogenic**	***L. interrogans***	**Australis**	**Pohnpei**	
5	**pathogenic**	***L. interrogans***	**Australis**	**Australis**	**Ballico**
6	**pathogenic**	***L. interrogans***	**Hebdomadis**	**Hebdomadis**	**Hebdomadis**
7	**pathogenic**	***L. interrogans***	**Icterohaemorrhagiae**	**Copenhageni**	**Wijnberg**
8	**pathogenic**	***L. interrogans***	**Djasiman**	**Djasiman**	**Djasiman**
9	**pathogenic**	***L. interrogans***	**Pyrogenes**	**Pyrogenes**	**NC-2016-001**
10	**pathogenic**	***L. interrogans***	**Pyrogenes**	**Manilae**	**LT 398**
11	**pathogenic**	***L. interrogans***	**Pyrogenes**	**Pyrogenes**	**Salinem**
12	**pathogenic**	***L. interrogans***	**Canicola**	**Canicola**	**Hond Utrecht IV**
13	**pathogenic**	***L. interrogans***	**Icterohaemorrhagiae**	**Icterohaemorrhagiae**	**Verdun**
14	**pathogenic**	***L. interrogans***	**Autumnalis**	**Autumnalis**	**Akiyami A**
15	**pathogenic**	***L. borgpetersenii***	**Tarassovi**	**Tarassovi**	**Mitis Johnson**
16	**pathogenic**	***L. borgpetersenii***	**Sejroe**	**Sejroe**	**M 84**
17	**pathogenic**	***L. borgpetersenii***	**Sejroe**	**Hardjobovis**	**Sponsele**
18	**pathogenic**	***L. borgpetersenii***	**Mini**	**Mini**	**Sari**
19	**pathogenic**	***L. borgpetersenii***	**Javanica**	**Javanica**	**Veldrat Bataviae 46^T**
20	**pathogenic**	***L. borgpetersenii***	**Ballum**	**Castellonis**	**Castellon 3**
21	**pathogenic**	***L. weilii***	**Celledoni**	**Celledoni**	**Celledoni^T**
22	**pathogenic**	***L. kirschneri***	**Grippotyphosa**	**Grippotyphosa**	**Moska V**
23	**pathogenic**	***L. kirschneri***	**Cynopteri**	**Cynopteri**	**3522 C**
24	**pathogenic**	***L. noguchii***	**Panama**	**Panama**	**CZ 214K^T**
25	**pathogenic**	***L. noguchii***	**Louisiana**	**Louisiana**	**LSU 1945**
26	**pathogenic**	***L. santarosai***	**Shermani**	**Shermani**	**1342 K**
27	pathogenic	*L. mayottensis*	Mini		200901116^T
28	pathogenic	*L. alexanderi*	Manhao	Manhao 3	L 60^T
29	pathogenic	*L. alstonii*		Sichuan	79601^T
30	pathogenic	*L. kmetyi*	Tarassovi	Malaysia	Bejo Iso 9^T
31	pathogenic	*L. kmetyi*			NC-JW3-C-A1
32	pathogenic	*L. ellisii* sp. nov.			NC-ATI7-C-A5
33	pathogenic	*L. barantonii* sp. nov.			NC-FH4-C-A1
34	pathogenic	*L. adleri* sp. nov.			NC-FH2-B-D1
35	intermediate	*L. inadai*	Lyme	Lyme	10^T
36	intermediate	*L. fainei*	Hurtsbridge	Hurtsbridge	BUT 6^T
37	intermediate	*L. wolffii*		Khorat	Khorat-H2^T
38	intermediate	*L. wolffii*			NC-FH2-C-A2
39	intermediate	*L. licerasiae*		Varillal	VAR 010^T
40	intermediate	*L. broomi*			5399^T
41	intermediate	*L. venezuelensis*			CML-U50^T
42	intermediate	*L. perolatii* sp. nov.			NC-FH1-B-B1
43	intermediate	*L. neocaledonica* sp. nov.			NC-ES4-C-A1
44	intermediate	*L. saintgironsiae* sp. nov.			NC-FH4-c-A2
45	intermediate	*L. haakeii* sp. nov.			NC-ATI7-C-A4
46	intermediate	*L. hartskeerlii* sp. nov.			NC-MCA2-B-A3
47	saprophyte	*L. idonii*	Hebdomadis		Eri-1^T
48	saprophyte	*L. wolbachii*		Codice	CDC^T
49	saprophyte	*L. vanthielii*		Holland	Waz Holland^T
50	saprophyte	*L. terpstrae*		Hualin	LT 11-33^T
51	saprophyte	*L.meyeri*	Semaranga	Semaranga	Veldrat Semarang 173
52	saprophyte	*L. meyeri*			NC-ATI2-C-A2
53	saprophyte	*L. yanagawae*	Semaranga	Sao Paulo	Sao Paulo^T
54	**saprophyte**	***L. biflexa***	**Semaranga**	**Patoc**	**Patoc I^T**
55	saprophyte	*L. harrisiae* sp. nov.			NC-FH2-B-A1
56	saprophyte	*L. johnsonii* sp. nov.			NC-MCA2-B-A1
57	saprophyte	*L. ellinghausenii* sp. nov.			NC-JW2-C-A2
58	saprophyte	*L. macculloughii* sp. nov.			NC-ATI2-C-A1
59		*Turneriella parva*			H^T
