# Supplementary material for: Biodiversity of Environmental Leptospira: Improving Identification and Revisiting the Diagnosis
Source: Front Microbiol. 2018 May 1;9:816. doi: 10.3389/fmicb.2018.00816 (PMC5938396; doi:10.3389/fmicb.2018.00816)
Supplement: Supplementary file 5 [file Table_2.PDF]

Supplementary Table 2. Cycle thresholds (Ct) obtained with the original (Stoddard et., 2009) and the modified primers

	lipL-32 0.2 ng Ct values		
	LipL32-45F LipL32-286R	LipL32-47Fd LipL32-301Rd	Ct changes
L. interrogans Verdun	24.2	24.37	+0.17
L. kirschneri Moska V	24.1	24.18	+0.08
L. noguchii CZ214 K^T	24.31	24.57	+0.26
L. santarosai 1342K	23.5	23.34	-0.16
L. borgpetersenii B3-13S	22.93	22.76	-0.17
L. weilii Celledoni^T	25.22	23.64	-1.58
L. alexanderi L60^T	41.81	24.87	-16.94
L. mayottensis 200901116^T	38.37	26.05	-12.32
L. alstonii 79601^T	33.11	26.05	-7.06
L. kmetyi Bejo Iso 9^T	31.2	24.48	-6.72
L. ellisii sp. nov.	31.19	28.37	-2.82
L. barantonii sp. nov.	29.66	28.44	-1.22
L. adleri sp. nov.	32.25	28.65	-3.6
