# Supplementary material for: Biodiversity of Environmental Leptospira: Improving Identification and Revisiting the Diagnosis
Source: Front Microbiol. 2018 May 1;9:816. doi: 10.3389/fmicb.2018.00816 (PMC5938396; doi:10.3389/fmicb.2018.00816)
Supplement: Supplementary file 6 [file Table_3.PDF]

Supplementary Table 3. Cycle thresholds (Ct) and melting temperatures (T_m) following amplification of *lfb1* (Merien et al., 2005) from 0.2ng pure DNA

	lfb-1	
	0.2 ng	
	Ct	T_m
L. interrogans Verdun	23.43	83.06
L. kirschneri Moska V	24.15	84.04
L. noguchii CZ214 K^T	24.93	84.11
L. santarosai 1342K	25.63	86.55
L. borgpetersenii B3-13S	26.17	85.74
L. weilii Celledoni^T	26.22	84.86
L. alexanderi L60^T	25.9	85.43
L. mayottensis 200901116^T	29.22	85.2
L. alstonii 79601^T	40.09	86.07
L. kmetyi Bejo Iso 9^T	38.04	86.64
L. ellisii sp. nov.	37.46	88.35
L. barantonii sp. nov.	47.48	86.53
L. adleri sp. nov.	44.69	86.3
